# A Unique Case of Hepatocellular Carcinoma Treated with Radiofrequency Ablation with More than 12 Years Overall Survival: A Case Report

**DOI:** 10.1155/2010/151846

**Published:** 2011-01-02

**Authors:** Loukas Thanos, Nikolaos Ptohis, Anastasia Pomoni, Evangelia Sotiropoulou, Maria Pomoni, Dimitrios Kelekis

**Affiliations:** ^1^Department of Computed Tomography and Interventional Radiology, “Sotiria” General Hospital of Chest Diseases, Mesogion Avenue 152, 11527 Athens, Greece; ^2^Research Centre of Radiology and Imaging, “Evgenidion” General Hospital, Papadiamantopoulou Street 20, 11528 Athens, Greece

## Abstract

The case of a 72-year-old male patient with HCC is presented in whom percutaneous RFA was used as the sole first-line anticancer treatment, since he denied having partial hepatectomy. The patient underwent RFA two more times, at 1.5 years for treating a local tumor progression at the initial ablation site and at 11 years after the first session for treating a new remote intrahepatic recurrence. He revealed a long-term survival of more than 12 years so far and still remains in excellent clinical status.

## 1. Introduction

Although surgical resection is the gold standard treatment of HCC, only a limited number of HCC patients are surgical candidates because of their lack of hepatic reserve, resulting from coexisting advanced cirrhosis, widespread intrahepatic involvement, and concomitant diseases [1]. Furthermore, the shortage of donors and the high cost of liver transplantation, limit its use. 

 RFA is a widely accepted alternative to surgical resection and plays a key role in the therapeutic management of early stage HCC [2]. However, selecting the treatment modality to suit individual patients with HCC remains a matter of debate [3].

## 2. Case Presentation

**Figure 1:**
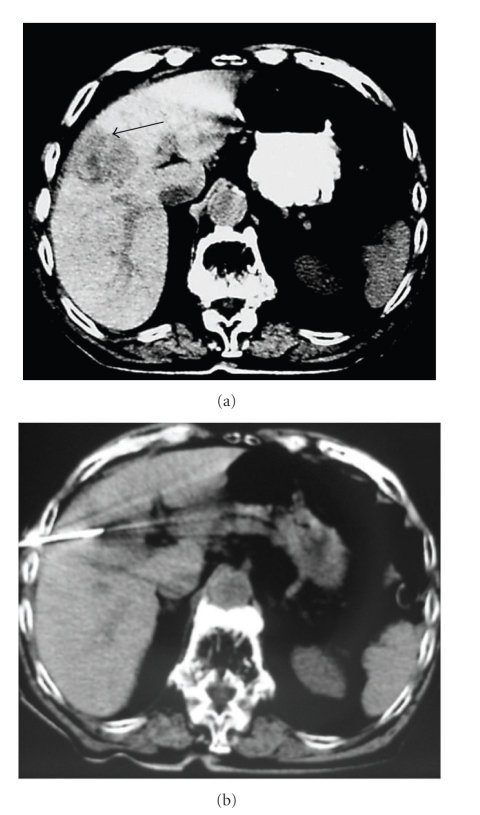
(a) A hypodense lesion with maximum diameter of 4.5 cm located in the IVth segment of the liver (arrow), with signs of early arterial enhancement, consistent with HCC characteristics. (b) The tip of the electrode is seen inside the lesion during the first RFA session.

A 72-year-old cirrhotic male patient, related to hepatitis C virus infection, presented about 12 years ago with elevation of a-fetoprotein (AFP) levels up to 110 ng/mL during routine laboratory tests. Computed tomography (CT) scan performed at that time demonstrated a hypodense lesion, 4.5 cm in maximum diameter, located in the IVth segment of the liver, with signs of early arterial enhancement consistent with HCC characteristics (Figure 1(a)). A biopsy was performed, and the histopathological results confirmed the diagnosis of HCC. In view of the fact that the patient had only a single lesion and was in functional grade A of Child-Pugh classification, he seemed to be a suitable candidate for surgical resection. At the same time, since he was categorized as having Class II Congestive Heart failure related to ischemic disease, he denied to undergo surgical treatment.

 Taking into consideration the tumor's size, location, and morphology, RFA seemed to be the most appropriate treatment. The whole procedure, which was performed percutaneously at the CT-scan suite, was explained to him, and an informed concept was obtained. The RFA was performed under local anesthesia. Forty-five minutes before the procedure, he received an analgesic and antidepressant medication, consisting of 0,05 gr pethidine hydrochloride intramuscularly and 3 mg bromazepam [Lexotanil Roche] per os for better patient's collaboration.

 Initially, a preprocedure CT scan was obtained. A cool-tip electrode was used [RADIONICS (Burlington, MA)] and a pulsed RF energy was applied for 15 min (Figure 1(b)). After the ablation of the lesion was completed, a low pulse RF energy was applied for the ablation of the track. To evaluate the immediate lesion's response to the ablation, a dual-phase dynamic contrast enhanced CT was performed after the electrode removal. Follow up was performed at 1, 3, and 6 months post-RFA and every 6 months afterwards.

 The patient did not experience any pain, and the postprocedural imaging findings revealed no enhancement. AFP levels presented significant reduction and finally normalization two weeks later. 

**Figure 2:**
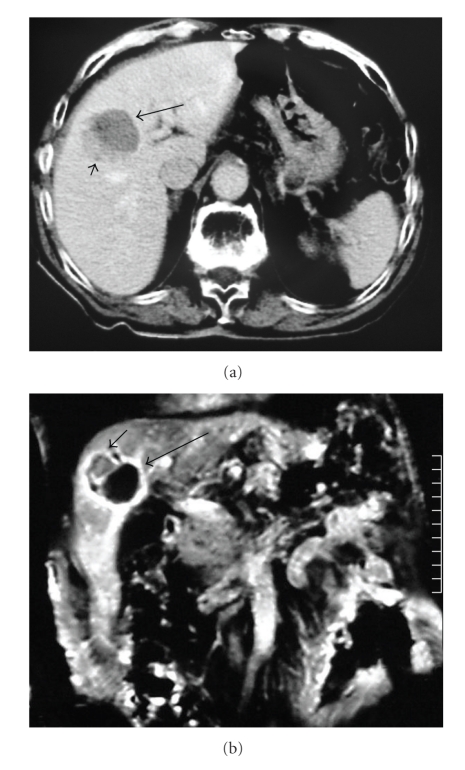
(a) After 18 months, a new CT scan depicted a local tumor progression at the ablation site. Note the unenhanced area of the lesion corresponding to the ablated site (long arrow) and the local tumor progression seen as an adjacent hypodense area (short arrow). (b) Similar to findings on the MRI scan, the ablated area shows no signal (long arrow), whereas the local tumor progression appears as a low signal area (short arrow).

 For a 18-month follow-up period the patient presented excellent clinical status with normal levels of AFP and stable appearance of the lesion on CT and magnetic resonance imaging (MRI) scans, when a new rise of the AFP levels to 80 ng/mL prompted us to repeat a new CT and MRI scan (Figures 2(a) and 2(b)); the CT images shown depict the local tumor progression as a slightly hypodense area adjacent to the unenhanced area of the lesion corresponding to the ablated site (short and long arrows, resp. on Figure 2(a)). Similar to the findings on the MRI scan (Figure 2(b)), the ablated area shows no signal (long arrow), whereas the local tumor progression appears as a low signal area (short arrow). A second RFA session, following the same protocol, was performed with technical success.

 For a 9-year follow-up period the patient presented excellent clinical status with normalization of AFP levels. Moreover, the tumor's maximum diameter decreased from 4.5 cm to 0.5 cm with well-defined margins mimicking a cystic formation on CT scans.

**Figure 3:**
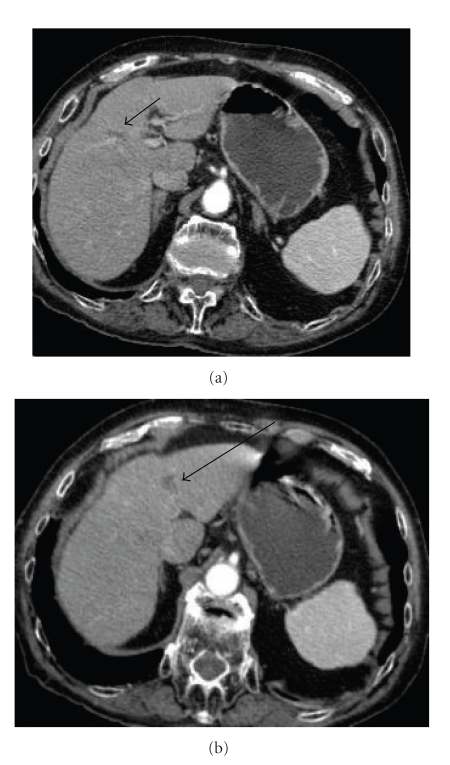
Latest CT scan, 12 years after the initial RF procedure; both lesions (arrows) are hardly visualized and might be misdiagnosed as cystic formations in the liver.

 Nine and half years after the second RFA, a new remote intrahepatic recurrence, located in the IInd segment of the liver, was suspected because of another increase of AFP values to 74 ng/mL during follow-up. RFA was once more the therapeutic option. A multitined expandable electrode was used [RITA StarBustTM XL electrosurgical device (RITA Medical Systems, Inc, Mountain View, California) 2-3 cm/15 cm length], and a pulsed RF energy was applied for 15 min. Hypoattenuating, nonenhancing areas were observed during both the arterial and portal venous phases, so the technique was considered to be completed. Overall, 12 years after the initial RF procedure, the patient was still alive with good quality of life and no imaging findings or elevation of AFP levels that may indicate a new recurrence. In the latest CT scan, performed at the same period (12 years after the initial RF procedure), both lesions were hardly visualized and could have been misdiagnosed as cystic formations in the liver (Figures 3(a) and 3(b)).

## 3. Discussion

 Accurate tumor staging at diagnosis is crucial for the therapeutic management of patients with HCC. According to Barcelona-Clinic Liver Cancer classification, patients at an early stage are those who present asymptomatic single HCC <5 cm or up to 3 nodules <3 cm. They will benefit from curative/effective therapies: resection, liver transplantation, and percutaneous ablation. In the case of single HCC <5 cm with signs of liver deficiency or in the case of 3 nodules <3 cm, RFA is the optimal treatment when other diseases coexist [4].

Additionally, the Child-Pugh score is used to assess the prognosis of cirrhosis, as well as the required strength of treatment and the necessity of liver transplantation. The score employs five clinical measures of liver disease. Each measure is scored 1–3, with 3 indicating most severe derangement (Table 1). Chronic liver disease is classified into Child-Pugh class A to C, employing the added score (Table 2).

 The therapeutic results of RFA of hepatic tumors are influenced by a variety of factors such as the skill of the operator, the choice of technique, the generator power of the RFA device, and the size, location, and morphology of the tumor [5, 6].

RFA uses high temperatures aiming at the destruction of the cancer cells. Alternating current through the tissue creates friction on a molecular level. Increased intracellular temperature generates localized interstitial heating. Temperatures between 60–100°C cause rapid denaturation and coagulation of the cellular proteins. Moreover, RFA seems to stimulate tumor-specific T lymphocytes and produce expression of heat sock proteins activating the process of apoptosis [7, 8]. Furthermore, the cool tip electrode allows internal circulation of water, which cools the tissue adjacent to the exposed electrode, maintaining low impedance during the treatment cycle. Low impedance permits maximum energy deposition for a larger ablation volume.

 Despite of the effectiveness of the technique, few reports that include long-term survival rates have been published [9–12]. In a large study consisting of 187 patients who were excluded from surgery and who had Child A or B cirrhosis, with either a single or multiple (as many as three) HCCs, less than or equal to 3 cm in diameter each, RFA was the first choice of treatment. The 3- and 5-year survival rates of patients with Child A cirrhosis were 76% and 51%, respectively, and for Child class B cirrhotic patients survival rates were 46% at 3 years and 31% at 5 years [9]. 

 Rossi et al. [11] reported 1-, 2-, 3-, 4-, and 5-year survival rates to be 94%, 86%, 68% 40%, and 40%, respectively. Furthermore, Buscarini et al. [12] reported an overall survival rate of 33% at 5 years. Disease-free survival at 5 years was 3%. Finally, Kim et al. [2] estimated 1-, 3-, and 5-year cumulative survival rates to be 95%, 64%, and 51%, respectively. Five-year cumulative survival rates for patients with HCC grade I, II and III were 71%, 44%, and 43%, respectively, with no statistical significance (*P* > .05). Four-year cancer-free survival rates in groups 1, 2, and 3 were 39%, 10%, and 0%, respectively.

 In our case although the overall survival could not have been predicted 12 years ago, our decision is now proving to have the most satisfactory result. At present, the ablated tumors are cystic and have a small size, evidence of a good response to RFA. During the 12 years period, the clinical status of the patient remained and remains still excellent. 

 Summarizing, taking into consideration our patient's survival and the fact that he remains still alive with good quality of life for a period of more than 12-years after the initial RF procedure, we may suggest that percutaneous RFA should be considered as an alternative means of treatment in the long-term management of hepatocellular carcinoma.

## Figures and Tables

**Table 1:** Child-Pugh score.

Measure	1 point	2 points	3 points
Total bilirubin, *μ*mol/l (mg/dl)	<34 (<2)	34–50 (2-3)	>50 (>3)
Serum albumin, g/l	>35	28–35	<28
INR	<1.7	1.71–2.20	> 2.20
Ascites	None	Mild	Severe
Hepatic encephalopathy	None	Grade I-II	Grade III-IV

**Table 2:** Interpretation of the Child-Pugh score.

Points	Class
5-6	A
7–9	B
10–15	C
